# Childhood adversity and cognitive impairment in later life

**DOI:** 10.3389/fpsyg.2022.935254

**Published:** 2022-08-16

**Authors:** Xiaoling Xiang, Joonyoung Cho, Yihang Sun, Xiafei Wang

**Affiliations:** ^1^School of Social Work, University of Michigan, Ann Arbor, MI, United States; ^2^School of Social Work, Columbia University, New York, NY, United States; ^3^School of Social Work, David B. Falk College of Sport and Human Dynamics, Syracuse University, New York, NY, United States

**Keywords:** childhood adversity, adverse childhood events, ACE, cognitive impairment, dementia, life course

## Abstract

**Objectives:**

This study examined the association between childhood adversity and cognitive impairment in later life and explored the potential moderation effect of gender and race.

**Methods:**

The study sample included 15,133 participants of the Health and Retirement Study (1998–2016 surveys) who had complete data on key study measures and were more than 50. The outcome variable is a dichotomous indicator of cognitive impairment as assessed by the Telephone Interview for Cognitive Status for self-respondents and the 16-item Informant Questionnaire on Cognitive Decline in the Elderly for proxies. A total of six childhood adversity indicators included grade retention, parental substance abuse, physical abuse, trouble with the police, moving due to financial hardship, and receipt of help due to financial hardship in early life. The estimation of the association between childhood adversity and cognitive impairment involved Cox proportional hazards regression. Results: Grade retention had the largest effect on incident cognitive impairment (HR = 1.3, 95% CI = 1.23–1.38, *p* < 0.001), followed by physical abuse by a parent (HR = 1.10, 95% CI = 1.00–1.20, *p* = 0.001). The impact of grade retention was more detrimental to women than men (interaction term HR = 0.89, 95% CI = 0.80–1.00, *p* = 0.048, female as the reference). Parental substance abuse was associated with a lower risk of incident cognitive impairment for most racial groups (HR = 0.89, 95% CI = 0.83–0.95, *p* = 0.001), but this association was reversed in “non-Hispanic other” race, consisting mainly of Asians (HR = 1.54, 95% CI = 1.05–2.26, *p* = 0.025).

**Discussion:**

Some aspects of childhood adversity continue to harm cognitive functioning in later life, while some events may have the opposite effect, with evidence of heterogeneity across gender and race.

## Introduction

Cognitive impairment and dementia are leading causes of disability and death worldwide and are a global health challenge in the context of rapid population aging (Nichols et al., 2019). A life course approach to chronic disease epidemiology suggests that the development of cognitive impairment is a dynamic process involving the interplay between psychosocial and biological risk factors over the life span (Lynch and Smith, 2005). Childhood, in particular, is regarded as a pivotal life stage leading to social inequality and, subsequently, differences in adult health. Childhood disadvantages, especially traumatic experiences, are theorized to affect adult health directly and indirectly through adult socioeconomic status, lifestyle choices, and psychosocial resources (Ferraro et al., 2016). An abundance of research has linked childhood disadvantages to an array of health conditions in adulthood, such as depression, anxiety, substance abuse, sexually transmitted diseases, chronic pain, musculoskeletal disorders, gastrointestinal issues, and overall health (Dube et al., 2003; Wade et al., 2016; Dobson et al., 2020; Riedl et al., 2020; Ross et al., 2020). A growing body of research suggests that age-related cognitive decline may originate in early life experiences, prompting recommendations for the early prevention of dementia (Livingston et al., 2020; Patel and Oremus, 2022).

Childhood adversity is an umbrella term to describe a broad set of life events and experiences that cause severe stress or overwhelm the capacity to cope during childhood, ranging from physical and sexual abuse to chronic poverty (Anda et al., 2006; McLaughlin et al., 2014). A plethora of research has associated childhood adversity indicators with a wide range of physical and mental health conditions, unhealthy behaviors and deficits in social relationships, and poor socioeconomic outcomes in adulthood (Hughes et al., 2017; Petruccelli et al., 2019). Animal and human studies have also linked adverse events experienced early in life to impaired cognitive development during childhood and adolescence. In animal studies, maternal deprivation causes the rearrangement of numerous brain structures and functions that have enduring effects in rats and monkeys (Lupien et al., 2009). Human studies have accumulated substantial evidence for a causal relationship between child maltreatment and reduced cognitive performance in institutionalized children (Young-Southward et al., 2020). In community samples, abused and neglected children frequently performed worse on a range of cognitive tests, including spatial working memory tasks (Augusti and Melinder, 2013), intelligence (Kočovská et al., 2012), measures of attention, visual-motor integration, and concept formation (Nolin and Ethier, 2007).

The effect of childhood adversity on cognitive function can be tracked across the life course through several biopsychosocial pathways. The most discussed biological pathway is the glucocorticoid cascade hypothesis, where early adversity evokes atypical development of the hypothalamic-pituitary-adrenal axis stress response, resulting in structural changes in several brain regions, atrophy of the hippocampus, and decreased activity of the prefrontal cortex, and subsequently, affecting learning and memory (Sapolsky et al., 1986; McCrory et al., 2010). Relatedly, early adversity could diminish cognitive reserve, a broad term referring to the adaptability related to the differential susceptibility of cognitive abilities to brain aging and pathology (Stern et al., 2020). Cognitive reserve has been linked to cognitive and functional outcomes later in life (Sardella et al., 2020). Diminished cognitive reserve due to childhood adversity could make individuals more susceptible to cognitive declines later in life (Fors et al., 2009). Exposure to adverse childhood conditions could also affect cognition through psychosocial pathways involving the development of self-esteem and coping strategies, socioeconomic disadvantages in adulthood (e.g., lower academic achievement and social class), health behavior patterns (e.g., smoking), and morbidity (e.g., cardiovascular disease) throughout the life course (Fors et al., 2009; Hughes et al., 2017; Petruccelli et al., 2019).

However, empirical evidence is unclear whether the detrimental impact of childhood adversity on cognition earlier in life persists into older adulthood, with conflicting findings in the extant literature. A British 1946 birth cohort study found a strong association between material and emotional indicators of childhood adversity and lower cognitive ability in childhood and adolescence, but little evidence that these adverse effects persisted over middle age (Richards and Wadsworth, 2004). However, studies using aggregate childhood adversity scales found an increased risk of incident dementia in older adults across cultural contexts (Donley et al., 2018; Tani et al., 2020). Early life food insecurity was also associated with a two-fold increase in the odds of dementia in pooled estimates from a meta-analysis of relevant studies (Wang et al., 2019). Findings are inconsistent among studies examining individual adverse childhood events and cognitive dysfunction (Patel and Oremus, 2022). While some studies reported a significant adverse effect on later-life cognitive performance, particularly in clinical samples of older adults with mental disorders (Kilian et al., 2018; Petkus et al., 2018; Wells et al., 2020), others reported weak or no associations between childhood adversity indicators and cognition (Kobayashi et al., 2020).

Furthermore, in several studies, exposure to some adverse events was associated with better cognitive function or a slower rate of cognitive decline in older adult subgroups (Ritchie et al., 2011; Barnes et al., 2012; Feeney et al., 2013). For example, a population-based study of Irish older adults showed participants with childhood sexual abuse had better global cognition, memory, executive function, and processing speed, despite having poorer mental health outcomes (Feeney et al., 2013). In another regional sample of community-dwelling older adults in France, parental loss was associated with poorer cognitive performance in later life. In contrast, physical or sexual abuse was associated with a lower risk of cognitive impairment (Ritchie et al., 2011). These findings suggest that the types and severity of adverse events may affect cognition differently.

Emerging evidence also suggests the differential impact of childhood adversity on cognition by gender and race (Ritchie et al., 2011; Zhang et al., 2016; Donley et al., 2018), which may explain the null and mixed findings reported in studies that did not consider moderation effects. Other possible causes of these conflicting findings are variations in study designs, sample characteristics, and childhood adversity measures. More high-quality studies with longitudinal designs are needed to clarify childhood adversity’s impact on cognition in later life (Wang et al., 2019).

This study aimed to investigate the association between childhood adversity and incident cognitive impairment in older adults and explore the potential moderation effect by gender and race in a population-based sample of US older adults. We hypothesized that adverse childhood events would be associated with a higher risk of incident cognitive impairment among older adults. The prospective design allowed us to identify long-term effects over 18 years. The population-based and representative samples reduced sample bias and improved generalizability. We included various adverse events covering different childhood environmental dimensions to uncover variations in association patterns. Life course scholars have called for examining multiple domains of childhood disadvantages simultaneously, given their interconnectedness (Ferraro et al., 2016). We further hypothesized that the extent of childhood adversity’s adverse impact on cognitive impairment is a function of gender and race.

## Materials and methods

### Data

Our study sample and data came from the Health and Retirement Study (HRS), a nationally representative study of people over age 50 in the United States, sponsored by the National Institute on Aging (NIA U01AG0097) and housed at the University of Michigan Institute for Social Research. HRS uses a national area probability sample of US households with supplemental oversamples of Blacks, Hispanics, and residents of the state of Florida. The majority of HRS sample population is approaching retirement or already retired, but individuals not in the labor force or who have never worked outside the home are also included (Heeringa and Connor, 1995). HRS employs a multistage area probability design with geographic stratification and clustering and a steady-state design that refreshes the sample every 6 years. Eligible participants undergo biennial interviews. More information about HRS study design and sample selection is available on its website.^1^

### Study sample

We analyzed data from the 1998 to 2016 interviews and included all available birth cohorts except for the Late Baby Boomers (added in 2016) due to limited follow-up data. Specifically, we used HRS Biennial Data Products 1998 through 2016 HRS Core files, and the RAND Contributed Files RAND HRS Longitudinal File 2016 (v.2). This study analyzed publicly available, de-identified data and was determined as “not regulated as human subjects research” by the University of Michigan Institutional Review Board.

The study sample included 15,133 HRS participants who met the following inclusion criteria: (a) had no missing data on the childhood adversity measures (described in the section below) and (b) ≥51 years at baseline. Study participants completed up to 10 interviews spanning over 18 years. The mean number of interviews completed was 7. By the end of the 18-year follow-up, about 30% of the study sample (*N* = 4,515) were non-responsive, including 3,057 who died.

### Measures

#### Cognitive impairment

The HRS administers cognitive tests to all self-respondents based on items from the Telephone Interview for Cognitive Status (TICS), a validated cognitive assessment designed for telephone surveys (Brandt et al., 1988). These tests included an immediate and delayed word recall test (0–20 points), a serial 7s test (0–5 points), and a backward counting test (0–2 points), summing up to a 27-point scale. Test administration and procedures are detailed in several HRS reports accessible *via* its website.^2^ Based on validation studies, a score of 0–6 indicated dementia, 7–11 indicated cognitive impairment without dementia (CIND), and 12 or over indicated normal (Crimmins et al., 2011; Langa et al., 2017). We combined the CIND and dementia into a single category of cognitive impairment to facilitate data analysis.

The HRS methodology involved proxies, often the spouse or partner of the sampled respondent, to answer questions on behalf of the sampled person who could not participate in the interview. Cognitive performance tests intended for the sampled person are inappropriate for proxy interviews. Instead, proxies responded to the 16-item Informant Questionnaire on Cognitive Decline in the Elderly (IQCODE) that asked about the sample person’s change in memory in the last 2 years (Jorm, 1994). Based on findings from a systematic review, we used a cut-off of 3.3 on the IQCODE to classify cognitive impairment (Quinn et al., 2014). We combined self-report and proxy cognition measures to create a binary cognitive impairment indicator for the primary analysis. A score of ≤11 on the TICS or ≥3.3 on the IQCODE indicated cognitive impairment.

#### Childhood adversity

The HRS included comprehensive assessments of childhood adverse events, but these measures were not administered consistently across interviews or the entire sample (Smith et al., 2017). Four items, available between 2006 and 2012, asked about traumas before age 18: (1) “Did you have to do a year of school over again?”; (2) “Did either of your parents drink or use drugs so often that it caused problems in the family?”; (3) “Were you ever physically abused by either of your parents?”; and (4) “Were you ever in trouble with the police?” (the last item was available between 2008 and 2012 only). We also included two items about childhood economic hardship from 1998 through 2016 core surveys. Participants checked whether the following applied when they were growing up, from birth to age 16: (1) “Did financial difficulties ever cause you or your family to move to a different place?” and (2) “Was there a time when you or your family received help from relatives because of financial difficulties?” For each item, we extracted data from all available interviews to generate an indicator of experiencing that even if a “yes” response was ever recorded for the relevant item.

#### Covariates

Time-invariant demographic characteristics included gender (female and male), race/ethnicity (non-Hispanic White, non-Hispanic African American, non-Hispanic other race or mixed, and Hispanic), and highest educational attainment (less than high school, high school or equivalent, some college but no degree, and a college degree). Time-varying socio-demographic variables included marital status (married/partnered/cohabiting, divorced/separated/widowed, and never married) and household net wealth (divided into quartiles). Time-varying health behavior, health status, and functioning covariates involved a binary indicator of current smoking (yes or no), an indicator of elevated depressive symptoms (a score of 3 or more on the 8-item Center for Epidemiologic Studies Depression Scale), and the count of chronic physical conditions (hypertension, diabetes, heart disease, stroke, lung disease, cancer, and arthritis).

### Statistical analysis

We performed descriptive statistics using data from the 2010 interview because all the birth cohorts included in this study had entered the HRS by 2010. We applied the 2010 HRS survey weights and design factors using a Taylor Series Linearization of the estimator to generate nationally representative estimates.

We estimated the unadjusted cumulative incidence function (CIF) of cognitive impairment. The CIF is more appropriate than the Kaplan–Meier curve in the presence of a competing-risk event, defined as an event that can preclude or alter the occurrence of the failure event of interest (Austin et al., 2016). In this study, death is a competing event because participants who died without ever having cognitive impairment are no longer at risk of developing cognitive impairment. For ease of analysis, we used the stcrreg followed by stcurve command in Stata to obtain the CIF of cognitive impairment stratified by each childhood adversity indicator without adjusting for other covariates.

The impact of childhood adversity on cognitive impairment, adjusted for other covariates, was examined in a Cox proportional hazards regression model. We did not rely on subdistribution methods because they may produce more biases than cause-specific hazard ratios (e.g., Cox model) in estimating the causal effects of covariates (Lesko and Lau, 2017). The exploration of the potential moderation effects of gender and race involved entering the relevant interaction terms into the model. We used age as the timescale because it is more appropriate for survival analysis with older adults (Lamarca et al., 1998). We specified the entry time as the age when the participant first joined this study.

### Sensitivity analysis

We tested the aforementioned Cox model to predict dementia, defined as a score of ≤6 on the TICS or ≥3.3 on the IQCODE, to check the robustness against a different operationalization of cognitive impairment. We also used the Fine-Gray competing risk model (Fine and Gray, 1999) to check the robustness in the presence of a competing risk. These analyses were conducted using Stata 15 SE Version (College Station, TX: StataCorp LP).

## Results

Nearly half of the study sample (*n* = 7,008 or 46%) met the criteria for cognitive impairment at some point during the study period. About half of the study sample reported at least one childhood adversity, with a mean count of 0.8 (SD = 1.06). As shown in Table 1, older adults with childhood adversity experience were more likely to be men, racial and ethnic minorities, and current smokers. They had a lower level of educational achievements and wealth and a higher rate of elevated depressive symptoms and more chronic physical conditions.

**TABLE 1:** Descriptive statistics of the study sample in 2010 stratified by childhood adversity status.

	All	Any childhood adversity		
Sample characteristics		No	Yes	*P*-value
Age in years	66.5 (65.9, 67.0)	67.1 (66.5, 67.7)	65.9 (65.3, 66.4)	<0.001
**Gender (%)**				<0.001
Female	53.2 (52.3, 54.0)	58.0 (56.5, 59.5)	48.2 (46.8, 49.6)	
Male	46.8 (46.0, 47.7)	42.0 (40.5, 43.5)	51.8 (50.4, 53.2)	
**Race/ethnicity (%)**				<0.001
White, non-Hispanic	81.7 (79.4, 83.8)	83.4 (81.3, 85.3)	79.9 (77.1, 82.4)	
African American, non-Hispanic	83.8 (7.4, 9.4)	7.8 (6.8, 8.9)	9.0 (7.9, 10.2)	
Other, non-Hispanic	2.9 (2.3, 3.6)	2.7 (2.0, 3.7)	3.1 (2.4, 3.8)	
Hispanic	7.1 (5.4, 9.2)	6.1 (4.7, 7.9)	8.1 (6.1, 10.6)	
**Education (%)**				<0.001
Less than high school	13.4 (12.1, 14.7)	10.8 (9.3, 12.5)	16.0 (14.7, 17.4)	
High school or equivalent	33.6 (32.3, 34.9)	31.3 (29.7, 33.0)	35.9 (34.3, 37.5)	
Some college but no degree	25.1 (24.0, 26.2)	25.2 (23.9, 26.7)	24.9 (23.2, 26.7)	
College degree	28 (26.1, 30.0)	32.7 (30.2, 35.2)	23.2 (21.3, 25.2)	
**Marital status (%)**				0.58
Married, partnered, or cohabiting	64.7 (63.4, 66.1)	65.1 (63.4, 66.8)	64.4 (62.9, 65.9)	
Divorced, separated, or widowed	28.8 (27.6, 29.9)	28.3 (26.9, 29.7)	29.3 (27.8, 30.9)	
Never married	6.5 (5.9, 7.2)	6.7 (5.6, 7.9)	6.3 (5.6, 7.2)	
Household net wealth (in 2010 $)	488,739 (457568, 519910)	564,360 (522570, 606150)	410,603 (373388, 447818)	<0.001
Current smoker (%)	13.6 (12.6, 14.7)	10.9 (9.8, 12.1)	16.4 (15.0, 17.9)	<0.001
Has elevated depressive symptoms (%)	20.5 (19.6, 21.4)	16.6 (15.6, 17.7)	24.5 (23.3, 25.7)	<0.001
Count of chronic physical conditions	1.8 (1.8, 1.8)	1.7 (1.6, 1.7)	1.9 (1.9, 2.0)	<0.001

The 2010 HRS survey weights were applied in estimates. “Any childhood adversity” refers to reports of any of the following childhood adversity items: (1) did a year of school over; (2) parental alcohol or drug abuse; (3) physical abuse by either parent; (4) ever in trouble with the police; (5) financial difficulties caused move; and (6) received help from relatives because of financial difficulties.

The most prevalent childhood adversity was parental alcohol/drug problems or parental substance abuse (19.2%), followed by financial difficulties that caused a move (16.6%), repeated a year of school or grade retention (16.5%), and receipt of help from relatives due to financial difficulties (14.5%). In bivariate comparisons, cognitive impairment was more prevalent among those who repeated a year of school, those who experienced parental substance abuse, and those who reported financial difficulties that caused a move during childhood (Table 2).

**TABLE 2:** Prevalence of cognitive impairment in 2010 by the experience of childhood adversity.

Childhood adversity items	All	% with cognitive impairment	*P*-value
**Did a year of school over before the age of 18**			<0.001
No	83.5 (82.5, 84.5)	14.7 (13.6, 15.7)	
Yes	16.5 (15.5, 17.5)	27.0 (25.1, 29.0)	
**Either parent drank or used drugs so often that it caused problems in the family before the age of 18**			0.017
No	80.8 (79.7, 81.9)	17.2 (16.0, 18.4)	
Yes	19.2 (18.1, 20.3)	14.6 (12.9, 16.5)	
**Physically abused by either parent before the age of 18**			0.756
No	91.1 (90.4, 91.9)	16.6 (15.5, 17.9)	
Yes	8.9 (8.1, 9.7)	17.1 (14.7, 19.8)	
**Ever in trouble with the police before the age of 18**			0.341
No	92.2 (91.6, 92.8)	16.8 (15.7, 18.0)	
Yes	7.8 (7.2, 8.4)	15.2 (12.5, 18.4)	
**Financial difficulties caused a move to a different place through the age of 16**			<0.001
No	83.4 (82.4, 84.4)	15.7 (14.7, 16.7)	
Yes	16.6 (15.6, 17.6)	21.8 (19.3, 24.5)	
**Received help from relatives because of financial difficulties through the age of 16**			0.399
No	85.5 (84.7, 86.3)	16.6 (15.5, 17.7)	
Yes	14.5 (13.7, 15.3)	17.4 (15.5, 19.5)	

The 2010 HRS survey weights were applied in estimates.

**FIGURE 1:**
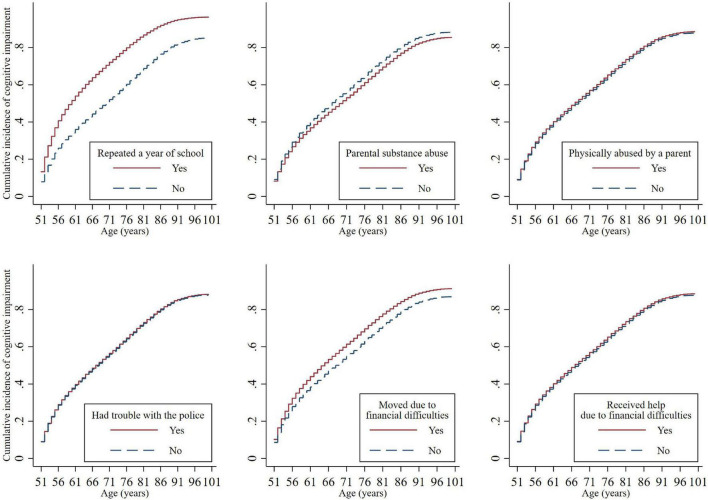
Cumulative incidence functions for cognitive impairment by childhood adversities. The cumulative incidence of cognitive impairment differed significantly by repeating a year of school (Subdistribution Hazard Ratio [SHR] = 1.74, 95% CI = 1.64–1.85, *p* < 0.001), parental substance abuse (SHR = 0.90, 95% CI = 0.84–0.96, *p* = 0.002), and financial difficulties that caused a move (SHR = 1.20, 95% CI = 1.12–1.27, *p* < 0.001).

Figure 1 presents the cumulative incidence of cognitive impairment with age, stratified by childhood adversity indicators. The cumulative incidence of cognitive impairment had a steeper increase over the lifetime for those who experienced some aspects of childhood adversity, including grade retention and moving due to financial difficulties. The cumulative incidence of cognitive impairment was slightly lower for individuals who experienced parental substance abuse. The unadjusted cumulative incidence did not differ for the remaining childhood adversity indicators.

In the adjusted analysis, individuals who repeated a year of school [Hazard Ratio (HR) = 1.30, 95% CI = 1.23–1.38, *p* < 0.001] and reported physical abuse by a parent (HR = 1.10, 95% CI = 1.00–1.20, *p* = 0.001) were more likely to have incident cognitive impairment as compared to those without the experience. Individuals with parental substance abuse experience were less likely to have incident cognitive impairment (HR = 0.89, 95% CI = 0.83–0.95, *p* = 0.001). The remaining childhood adversity indicators, including trouble with the police and two childhood poverty indicators, were not significantly associated with cognitive impairment in later life (Table 3).

**TABLE 3:** Results from Cox proportional hazard model on cognitive impairment.

Predictors	Hazard Ratio (95% CI)	*P*-value
**Gender**		
Female	Reference	
Male	1.19 (1.13, 1.26)	< 0.001
**Race/ethnicity**		
White, non-Hispanic	Reference	
African American, non-Hispanic	2.26 (2.12, 2.41)	< 0.001
Other, non-Hispanic	1.70 (1.47, 1.98)	< 0.001
Hispanic	1.68 (1.55, 1.81)	< 0.001
**Education**		
Less than high school	Reference	
High school or equivalent	0.54 (0.50, 0.57)	< 0.001
Some college but no degree	0.41 (0.38, 0.44)	< 0.001
College degree	0.27 (0.25, 0.29)	< 0.001
**Marital status**		
Married, partnered, or cohabiting	Reference	
Divorced, separated, or widowed	1.02 (0.96, 1.07)	0.606
Never married	1.01 (0.89, 1.15)	0.847
**Household net wealth in quartiles**		
1st quartile (bottom 25%)	Reference	
2nd quartile	0.76 (0.71, 0.81)	< 0.001
3rd quartile	0.63 (0.59, 0.68)	< 0.001
4th quartile (top 25%)	0.52 (0.48, 0.57)	< 0.001
Current smoker	1.23 (1.15, 1.31)	< 0.001
Has elevated depressive symptoms	1.29 (1.23, 1.36)	< 0.001
Count of chronic physical conditions	1.01 (0.99, 1.03)	0.257
**Childhood adversities**		
Did a year of school over	1.30 (1.23, 1.38)	< 0.001
Parental alcohol or drug abuse	0.89 (0.83, 0.95)	0.001
Physical abuse by either parent	1.10 (1.00, 1.20)	0.041
Ever in trouble with the police	0.90 (0.81, 1.00)	0.060
Financial difficulties caused moving	1.03 (0.96, 1.09)	0.407
Received help from relatives because of financial difficulties	0.99 (0.92, 1.06)	0.711

**FIGURE 2:**
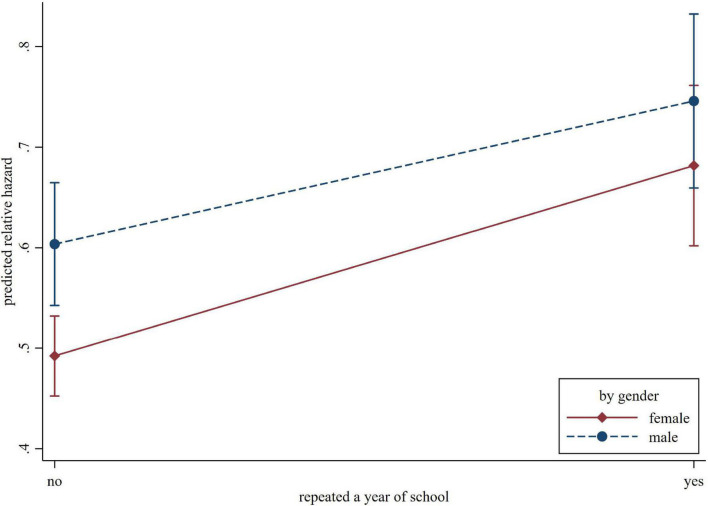
The predicted relative hazard of cognitive impairment concerning repeating a year of school, by gender.

The interaction terms between each of the potential moderators and childhood adversity indicators were entered one at a time. The interaction term between gender and grade retention was statistically significant (HR = 0.89, 95% CI = 0.80–1.00, *p* = 0.048), suggesting the impact of grade retention on cognitive impairment in later life was smaller for men than women (illustrated in Figure 2).

**FIGURE 3:**
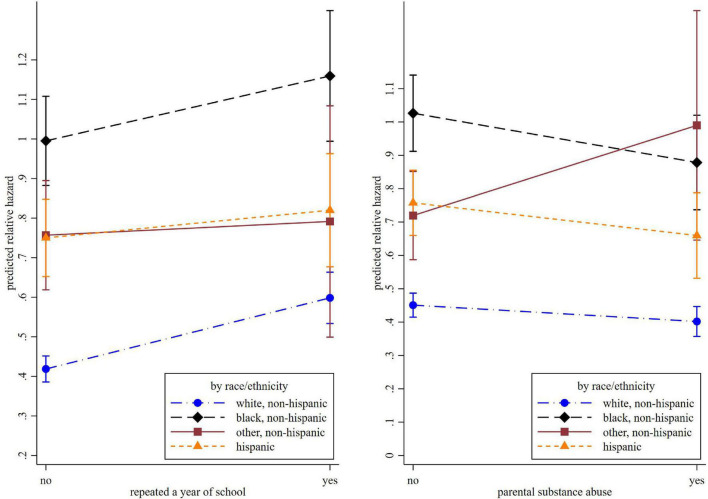
The predicted relative hazard of cognitive impairment concerning repeating a year of school and parental substance abuse, by race.

The association of grade retention and incident cognitive impairment also differed by race. The interaction terms “African American*grade retention” (HR = 0.82, 95% CI = 0.71–0.94, *p* = 0.004) and “Hispanic*grade retention” (HR = 0.76, 95% CI = 0.65–0.91, *p* = 0.002) were both statistically significant, suggesting an attenuated effect for these minority groups. Another significant interaction term is between non-Hispanic other race and parental substance abuse (HR = 1.54, 95% CI = 1.05–2.26, *p* = 0.025). In comparison, parental substance abuse was associated with a lower risk of cognitive impairment in other racial groups (HR = 0.89, 95% CI = 0.82–0.97, *p* = 0.006). These racial differences are illustrated in Figure 3.

### Sensitivity analysis

Estimates from the Cox regression predicting dementia were attenuated but in the expected direction. Repeating a year of school was associated with a higher risk of incident dementia (HR = 1.17, 95% CI = 1.05–1.30, *p* < 0.01). The impact of parental substance abuse (HR = 0.98, 95% CI = 0.86–1.11, *p* = 0.729) and physical abuse (HR = 1.02, 95% CI = 0.86–1.22, *p* = 0.798) were no longer statistically significant. Estimates from the fine-Gray competing risk model predicting cognitive impairment were similar to the Cox model within a difference of up to 0.04 on the exponentiated coefficients.

## Discussion

In a nationally representative sample of US older adults, the extent to which exposure to childhood adversity affected incident cognitive impairment was conditional on the type of adverse events, gender, and race. Out of the six adverse childhood events, grade retention had the most considerable impact on cognitive impairment; it was more detrimental to women than men and non-Hispanic whites than racial minorities. Incident cognitive impairment also increased for those exposed to parental physical abuse, and this effect did not differ by gender or race. Parental substance use, unexpectedly, was associated with a lower risk of cognitive impairment for most racial groups except for the “non-Hispanic other race” group (e.g., Asian, American Indian, and Alaska native).

Exposure to some aspects of childhood adversity continued to harm later-life cognitive status, supporting a life-course approach to understanding cognitive impairment etiology. Removing adult factors from the models yielded bigger effect sizes, suggesting a proportion of the effect was mediated through adulthood conditions. Specifically, after removing adulthood socioeconomic, health, and mental health status from the model, the hazard ratio concerning grade retention grew to 1.64 from 1.30, representing a 26% increase. Adding lifetime educational attainment back to the model reduced the hazard ratio from 1.64 to 1.35, suggesting that adverse educational experiences earlier in life lowered lifetime educational achievement, increasing the risk for poor cognitive function in later life. Adjusting for gender and race only, the hazard ratio concerning moving due to financial difficulties also increased and became statistically significant (HR = 1.09). This effect went away when adult household assets were considered. These findings are consistent with several recent studies (Donley et al., 2018; Petkus et al., 2018; Fu, 2019; Tani et al., 2020). As Fors et al. (2009) discussed, these similarities suggest “robust, universal mechanisms” underlying the association between childhood adversity and cognition in later life. These findings also align with the life course approach to health, which suggests that early exposure to disadvantages affects health indirectly through adult socioeconomic status, lifestyle choices, and psychosocial resources (Ferraro et al., 2016).

We found that women were more susceptible to the adverse effect of grade retention on cognitive impairment in later life. Previous studies have not documented a gendered effect of grade retention on cognitive impairment in later life to our best knowledge. Nevertheless, several studies have found a gendered effect of other types of childhood adversity (e.g., early parental loss, physical abuse) on cognition. For example, Ritchie et al. (2011) found that early parental loss was associated with poorer performance on a verbal test among older women but not older men. A recent study of older Japanese found that physical abuse was associated with dementia in men, whereas psychological neglect and abuse were associated with dementia in women (Tani et al., 2020). The Japanese study also reported a gendered dose-response relationship. Each increase in childhood adversity was associated with a higher dementia incidence among women but not among men (Tani et al., 2020). The exact mechanisms underlying these gendered effects are unclear. One possible pathway involves the adverse consequences of childhood adversity on physical and mental health in adulthood, which tend to be more detrimental for women than for men (Haatainen et al., 2003; Thompson et al., 2004). Another potential mediator relates to career achievement. Grade retention has been linked to poor educational career and eventual labor market outcomes (Eide and Showalter, 2001). Women who experienced grade retention may face the double jeopardy of grade retention and gender inequality and subsequently have worse educational and career outcomes. Although we adjusted for lifetime educational attainment in the analysis, we did not measure occupation type and complexity. Occupation attributes have been linked to the location of brain tissue loss or dysfunction in patients with frontotemporal dementia (Spreng et al., 2010). Jobs that require complex social interaction and thinking may increase cognitive reserve and protect against Alzheimer’s disease (Boots et al., 2015). In addition, grade retention can negatively affect self-concept aspects among children, and these adverse effects may be more pronounced for girls (Mathys et al., 2019). Finally, the gendered effects observed in our study could reflect the presence of survival bias. Men with a deprived childhood environment may be less resistant to adversities later in life and hence die at younger ages (Tani et al., 2020). As a result, we could have underestimated the association between grade retention and cognitive impairment among men.

Unexpectedly, parental substance abuse was associated with a lower risk of incident cognitive impairment for most racial and ethnic groups, including whites, African Americans, and Hispanics. This finding aligns with a previous study that found a better capacity for problem-solving, abstraction, and planning among neglected children (Nolin and Ethier, 2007). Similarly, the number of childhood maltreatment types was positively associated with cognition among preschool-aged foster children (Pears and Fisher, 2005). These findings suggest the possibility of resilience and post-traumatic growth. Resilience is the ability to use cognitive processing to achieve successful developmental and adaptive outcomes even in an extreme environment (Meschke and Patterson, 2003). Some children growing up in families with substance abuse problems can adapt and achieve better-than-expected outcomes (Velleman and Tempeton, 2016). Resilience is associated with many successful aging indicators and may confer cognitive benefits in later life (Wagnild, 2003). Another possible explanation is cohort and period effects. Drug use, particularly marijuana and psychedelic drug consumption, was one essential component of the Hippie counterculture in the 1960s, where drug use was viewed as “self-exploration, transcendental experiences, and spiritual enlightenment” (Wesson, 2011). Along with the anti-Vietnam War movement, the civil rights movement, and the sexual revolution, counterculture might be endorsed by a large number of baby boomers who were teenagers in the 1960s, so using drugs may not be attached with a negative social meaning in this age cohort.

However, the relationship between parental substance abuse and cognition was reversed for the “non-Hispanic other” group, which consisted mainly of Asian Americans. The positive association between parental substance abuse and cognitive impairment in later life among Asian Americans may be due to chance. The small sample size for the “non-Hispanic other” group reduces the reliability of the parameter estimate and increases variability. Cultural difference are also a plausible explanation. More than half of Asian Americans are foreign-born and may not have been subjected to the same period effects of the 1960s movements as native-born individuals. To illustrate, Chinese-origin Asian Americans are the US’s largest single Asian origin group. Rates of substance use in China have been relatively low since the founding of the People’s Republic of China in 1949, thanks to harsh punitive measures, including the death penalty, for drug-related crimes (McCoy et al., 1997). The new Chinese government was eager to implement extreme measures never to repeat the nation’s defeat during the Opium Wars and to control the epidemic of opioid addiction in the aftermath of the Opium Wars. The stigma associated with drug use is still prevalent in China, contributing to drug users’ self-stigma (Mak et al., 2015). Therefore, parental substance abuse may carry a more severe stigma and social and health consequences for Asian Americans than their non-Asian counterparts. It is worth pointing out that Asian Americans are underrepresented in the literature on health disparities, and very little research has focused on the cognitive health of older Asian Americans (Tang et al., 2019). Studies focused on other racial minority groups reported inconsistent findings. In one study, the association between cumulative childhood adversity and cognitive impairment was stronger among older African Americans than among whites (Zhang et al., 2016). However, in another study, food insecurity in childhood was associated with a slower cognitive decline rate in African Americans than in whites (Barnes et al., 2012). Our finding that Asians may respond to parental substance abuse in fundamentally different ways than other racial groups warrants more research and calls for more attention to the cognitive health of Asian Americans.

These study findings should be interpreted with some caution. All measures were self-reported and subject to recall bias and reporting errors. Despite its good psychometric properties, the TICS does not assess the full range of cognitive domains nor produce a clinical diagnosis. By including a set of adulthood conditions as covariates, our analyses may have an over-adjustment bias, as these factors can mediate the relationship between childhood adversity and later-life cognition (Ritchie et al., 2011). As discussed, we found partial and complete mediation related to early life educational experience and financial hardship through adult educational attainment and wealth accumulation. These overadjustments likely resulted in underestimates of the effects of childhood adversity on cognitive impairment. Nevertheless, we retained the model with more adjustments to reduce potential bias due to competing risks, which can be resolved only by including common risk factors as covariates (Lesko and Lau, 2017). Finally, we could not pinpoint the specific period when each adverse event occurred other than before age 18. Exposure to adverse events during different life stages (e.g., *in utero*, infancy, adolescence) may differently affect later-life outcomes (Lupien et al., 2009). These subtle effects remained hidden due to data limitations.

## Conclusion

Some aspects of childhood adversity continue to harm cognitive functioning in later life. In contrast, some events may have the opposite effect, with evidence of heterogeneity across gender and race. Future inquiries into the long-term impact of childhood adversity on cognition should consider both individual adverse events and aggregate scores of adverse events. More research is needed to explore the mechanisms and mediating factors underlying the gender and racial disparities in associations between early life experiences and cognition in later life.

## Data availability statement

Publicly available datasets were analyzed in this study. This data can be found here: https://hrs.isr.umich.edu/about.

## Ethics statement

Ethical review and approval was not required for the study on human participants in accordance with the local legislation and institutional requirements. The patients/participants provided their written informed consent to participate in this study.

## Author contributions

XX formulated the research question, designed the study, analyzed the data, and drafted most parts of the manuscript. JC and YS drafted parts of the manuscript and provided edits and feedback on the initial draft. XW advised on the construction of measurements and provided critical feedback on the initial draft. All authors contributed to the article and approved the submitted version.

## Footnotes

1
https://hrs.isr.umich.edu/documentation/survey-design

2
https://hrs.isr.umich.edu/documentation/user-guides
